# The novel automated peak frequency annotation algorithm for identifying high frequency electrogram activity following pulmonary vein isolation in atrial fibrillation ablation

**DOI:** 10.1093/europace/euae114

**Published:** 2024-05-06

**Authors:** Ming-Jen Kuo, Li-Wei Lo, Yenn-Jiang Lin, Steven Kim, Shih-Ann Chen

**Affiliations:** Division of Cardiology, Department of Medicine, Taipei Veterans General Hospital, No. 201, Sec. 2, Shipai Rd., Beitou, Taipei, 11217 Taiwan, R.O.C; Institute of Clinical Medicine and Cardiovascular Research Institute, National Yang-Ming Chiao-Tung University, No. 155, Sec. 2, Linong Street, 112 Taipei, Taiwan; Cardiovascular Center, Taichung Veterans General Hospital, 1650 Taiwan Boulevard Sect. 4, Taichung, 407219 Taiwan, R.O.C; Division of Cardiology, Department of Medicine, Taipei Veterans General Hospital, No. 201, Sec. 2, Shipai Rd., Beitou, Taipei, 11217 Taiwan, R.O.C; Institute of Clinical Medicine and Cardiovascular Research Institute, National Yang-Ming Chiao-Tung University, No. 155, Sec. 2, Linong Street, 112 Taipei, Taiwan; Division of Cardiology, Department of Medicine, Taipei Veterans General Hospital, No. 201, Sec. 2, Shipai Rd., Beitou, Taipei, 11217 Taiwan, R.O.C; Institute of Clinical Medicine and Cardiovascular Research Institute, National Yang-Ming Chiao-Tung University, No. 155, Sec. 2, Linong Street, 112 Taipei, Taiwan; Advanced Applications Department, Abbott, Plymouth, MN, USA; Division of Cardiology, Department of Medicine, Taipei Veterans General Hospital, No. 201, Sec. 2, Shipai Rd., Beitou, Taipei, 11217 Taiwan, R.O.C; Institute of Clinical Medicine and Cardiovascular Research Institute, National Yang-Ming Chiao-Tung University, No. 155, Sec. 2, Linong Street, 112 Taipei, Taiwan; Cardiovascular Center, Taichung Veterans General Hospital, 1650 Taiwan Boulevard Sect. 4, Taichung, 407219 Taiwan, R.O.C; National Chung Hsing University, 145 Xingda Rd., South Dist., 402 Taichung, Taiwan

**Keywords:** Atrial fibrillation, Pulmonary vein isolation, Peak frequency, Bipolar voltage, Conduction gap

## Introduction

Pulmonary vein (PV) isolation (PVI) is crucial for atrial fibrillation (AF) ablation. However, the presence of residual potentials along the circumferential ablation line during and after achieving PVI is known to contribute to long-term AF recurrence.^1–3^ Nevertheless, bipolar mapping has limitations in detecting residual potentials, particularly along the PV ablation line. A large peak-to-peak electrogram (EGM) may be attributed to a far-field component rather than local potential,^4^ and a small peak-to-peak EGM with high frequency may still indicate local myocardial activity.

In this study, a novel automated peak frequency (PF) algorithm, designed to identify high frequencies in each EGM, was evaluated for its effectiveness in detecting PV conduction gaps and dormant PV gaps following circumferential PV ablation.

## Method

The present study is a retrospective cohort study including 108 patients who received a catheter ablation for paroxysmal AF using Advisor™ HD Grid mapping catheter (Abbott, MN, USA) (IRB-TPEVGH number: 2023-08-003CC).

This study was conducted in two parts: in the phase 1 study, conduction gaps (GAP_active_) identified by left atrial (LA)-PV voltage and activation maps were acquired during sinus rhythm (SR) after first-pass circumferential ablation. The accuracy between voltage (V) and PF criteria to discriminate PV conduction gaps after first-pass PV circumferential ablation was compared, and optimal cut-off values for both the bipolar and omnipolar modalities of V and PF. Subsequently, in the phase 2 study with a population other than the phase 1 study, upon confirmation of PVI, an LA-PV high-density voltage map was created during SR again, enabling offline assessment of sites of post-PVI residual activity around the circumferential ablation line (GAP_dormant_). Applying the cut-off values established in the phase 1 study to the post-PVI LA-PV maps, we compared the relationship between the identification of sites of GAP_dormant_ based on various criteria during the index procedures and GAP_redo_ sites. The GAP_redo_ refers to the locations of conduction gaps during redo procedures in patients experiencing AF recurrence.

The procedural protocols have been described in our previous studies.^5–8^ The EnSite OT Near Field™ (OTNF) is a proprietary algorithm available within the Ensite X mapping system (Abbott, Abbott Park, IL), and the details were reported in the previous study.^9^ In brief, OTNF automatically tracks the highest frequency signal components along the time course of each intracardiac EGM, resulting in a unique PF trace (PF_t_). The PF_t_ is a wavelet-derived frequency function in which the signal is decomposed into short-time oscillations, based upon scaled and time-shifted functions of a time-localized mother wavelet.

Apart from automated absolute dV/dt_max_ annotation that is applied during daily practice, PF does not necessarily reflect the highest amplitude component of the EGM, but correlates with visual sharpness in EGM morphology, even at low amplitudes.

Sensitivity (SE), specificity (SP), positive predictive value (PPV), and negative predictive value (NPV) were calculated from the contingency table. A receiver operating characteristic (ROC) curve analysis was performed for different criteria in identifying the conduction gap and conduction block. The area under the ROC curve (AUC) was calculated as a measure of diagnostic accuracy. From the ROC analysis, optimal cut-off values were calculated using the Youden Index. The data were analysed using IBM SPSS Statistics (SPSS Inc., Chicago, IL, USA).

## Result

In the phase 1 study, a total of 50 GAP_active_ were identified over circumferential PVI lines in 20 patients. In the ROC curve analysis, both PF_bi_ and PF_omni_ demonstrated a significantly higher AUC in discriminating between conduction gaps and conduction blocks (for the V_bi_, V_omni_, PF_bi_, and PF_omni_, SE, SP, and AUC: 84.1, 75.0, 0.867; 66.5, 86.7, 0.875; 95.0, 96.9, 0.964; 97.7, 96.7, 0.973, respectively). The cut-off values for conduction gaps were determined as 0.20 mV, 0.32 mV, 190.25 Hz, and 222.82Hz for the V_bi_, V_omni_, PF_bi_, and PF_omni_, respectively.

**Figure 1:**
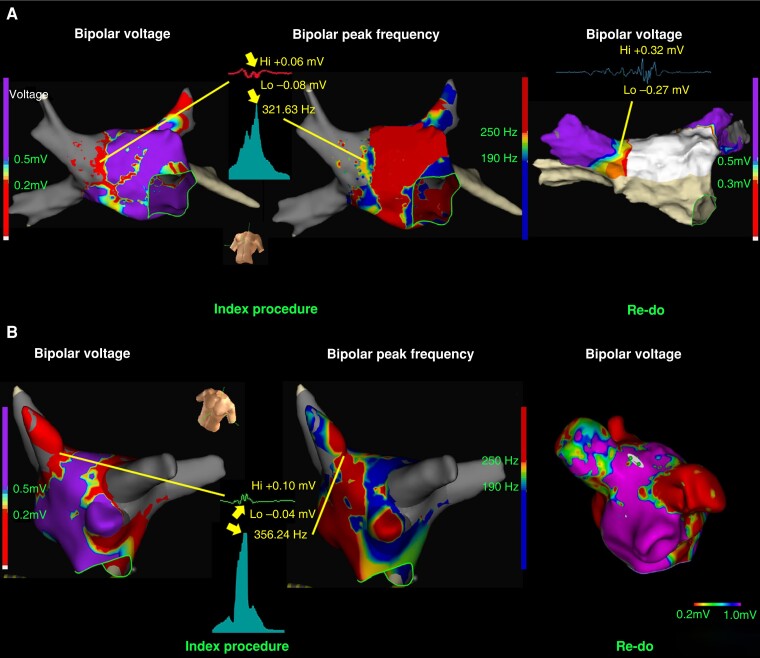
Representative redo cases of patients with AF recurrence and the comparison to the index procedures. In Panel *A*, there was a low amplitude of bipolar peak-to-peak voltage with voltage < 0.10 mV over the right anterior superior (RAS) segment of the circumferential pulmonary vein (PV) ablation line; however, after applying all the electrograms to peak frequency criteria, it showed high peak frequency activity over the corresponding region, besides, the potentials inside the right superior PV (RSVP) also showed high peak frequency value that was not observed by voltage criteria. During the redo procedure 6 months after the index ablation, potentials were found inside RSVP and the conduction gap was found over the RAS segment. Further segmental ablation over the RAS segment resulted in electrical isolation inside the RSVP. In Panel *B*, there was no anatomical gap found over the right superior (RS) segment of the circumferential PV ablation line by the voltage criteria; however, high residual activity was found by peak frequency criteria over the RS segment of the circumferential PV ablation line, in addition, potentials inside the RSVP also presented with high peak frequency activity. Eight months after the index procedure, potentials were found inside the RSVP by voltage criteria and the conduction gap was over the roof of the RSVP, compatible with the finding during the index procedure by peak frequency application.

In the phase II study, a study population of 88 patients were analysed (63 males, mean age of 57.7 ± 9.8 years). During a mean follow-up period of 12.7 ± 4.5 months, AF recurrences were observed in 26 patients (29.5%) following the index ablation procedures. Twelve patients underwent a redo procedure, and 37 GAP_redo_ were identified. When comparing the GAP_dormant_ detected during the index procedures using different criteria with the GAP_redo_ found during the redo procedures, both the voltage and PF criteria exhibited similar SP and NPV. As displayed in *Table 1*, the PF criteria demonstrated higher SE and PPV compared to the voltage criteria for predicting the presence of GAP_redo_. *Figure 1* presents cases between GAP_dormant_ during index procedure and the GAP_redo_ found during the redo procedures with both voltage and PF criteria.

**Table 1:** Accuracy of the GAP_dormant_ found by different criteria from the index procedure to the conduction gaps (GAP_redo_) found during the redo procedure

	PV segments with gaps (*n* = 37)			
	Sensitivity	Specificity	PPV	NPV
Bipolar peak-to-peak voltage	16.2%	96.1%	50.0%	82.8%
Omnipolar peak-to-peak voltage	10.8%	97.4%	50.0%	82.1%
Bipolar peak frequency	51.4%	96.8%	79.2%	89.3%
Omnipolar peak frequency	54.1%	95.5%	74.1%	89.7%

## Discussion

The intracardiac bipolar EGM recordings comprise a combination of near-field (NF) and far-field (FF) potentials. Annotating local activation times to the FF potentials could lead to misleading activation maps; on the contrary, if tiny signals are indiscriminately regarded as FF, it may overlook tissues that still exhibit electrical activity. In the present study, despite GAP_dormant_ detected over the circumferential PV ablation line in the index procedure exhibiting a good correlation with the GAP_redo_ (similar SP and NPV between V and PF), PF criteria improved the SE and PPV compared to voltage criteria. We demonstrated that potentials with low voltage but high PF may still represent true local electrical activity, which could contribute to future LA-PV conduction.

While the sharpness of the EGM can be gauged visually, the subjective nature of this approach may lead to considerable variability among electrophysiologists. Furthermore, during the AF ablation procedure, annotating the PF of each EGM offers a simple and intuitive approach to determining the presence of residual activity over the circumferential PV ablation line. Even if PVI is achieved, the presence of any potentials identified by the PF map after PV ablation may be indicative of future AF recurrence.

## Conclusion

In comparison to voltage criteria, the novel automated PF algorithm complements established mapping criteria and holds the potential to enhance the accuracy of LA mapping during AF ablation. Within our study series, utilizing PF, as opposed to voltage criteria, proves to be more effective in identifying residual activity along circumferential PV ablation lines during AF ablation procedures.

## Data Availability

The data underlying this article will be shared on reasonable request to the corresponding author.
